# The Journey of Acromegaly Towards Treatment: A Single-Center Study

**DOI:** 10.3390/jpm16020085

**Published:** 2026-02-02

**Authors:** Varvara Chalmantzi, Sophia Vlachou, Maria Eleni Chondrogianni, Maria Panagaki, Ariadni Spyroglou, Marina Tsoli, Eva Kassi, Gregory Kaltsas, Krystallenia I. Alexandraki

**Affiliations:** 1 Unit of Endocrinology, First Department of Internal Medicine, Laikon Hospital, 11527 Athens, PC, Greece; chalmantzi@gmail.com; 2 First Department of Propaedeutic Internal Medicine, Laikon Hospital, National & Kapodistrian University of Athens, 11527 Athens, PC, Greece; sophie-vl@hotmail.com (S.V.); marypanag23@gmail.com (M.P.);; 3 Second Department of Surgery, Aretaieio Hospital, National and Kapodistrian University of Athens, 11528 Athens, PC, Greece

**Keywords:** acromegaly, IGF-1, adenoma, biochemical control, elderly, comorbidities, neoplasia

## Abstract

**Background:** In the era of personalized medicine, the overall therapeutic approach has progressed throughout the years in acromegaly, but biochemical control of the disease is not achieved in a significant proportion of patients. This study aims to systematically record the journey of patients with acromegaly in the context of adenomas characteristics, therapeutic approaches and comorbidities in acromegaly with an emphasis in elderly. **Method:** In this retrospective study 79 patients were diagnosed with acromegaly between 1971 and 2023. **Results:** The dataset consisted of 43 (54%) female and 36 male (46%) with an overall mean age ± SD at diagnosis at 45 ± 13 years. 57 (73%) underwent one surgical procedure. Medical treatment with one agent was reported in 36 patients (67%), almost all by somatostatin analogs (89%). Radiotherapy was offered in 14 patients (18%). Disease remission was documented in 67 (85%) patients. IGF1/ULN at diagnosis displayed a tendency to predict non-remission. A diagnostic delay of less than five years was reported in 28 cases (65%) and patients reporting longer delays were older at diagnosis (58 ± 6 years). Patients diagnosed at or above the age of 60 were less likely to undergo a surgical procedure compared to patients diagnosed before the age of 60. **Conclusions:** Biochemical control was the most frequent disease outcome. A higher IGF-1/ULN ratio tends to predict non-remission. Longer diagnostic delay was reported with advancing age and older patients were less likely to follow surgical procedures.

## 1. Introduction

Acromegaly is a rare disease [1], usually diagnosed in the fifth decade of life with a diagnostic delay of about 5 years [1], which arises due to growth hormone (GH) hypersecretion most frequently caused by a GH-secreting pituitary adenoma [2]. This condition deregulates the growth pattern in several tissues thus gradually altering a patient’s appearance [3]. Abnormally elevated GH and insulin-like growth factor-1 (IGF-1) levels affect several systems and lead to a series of chronic complications, thus burdening the cardiometabolic, respiratory, and musculoskeletal patient profile [4]. If left untreated, life expectancy can be reduced by almost a decade [2]. In the past, mortality was attributed to cardiovascular disease [3]; nevertheless, nowadays cancer is the leading cause of death [4].

The diagnosis is established in patients who present with typical signs of the disease and IGF-1 levels over one to three times the upper limit of normal (ULN) for their age, while the main therapeutic target is maintaining IGF-1 levels within the normal range respective to the age of the patient [1].

Surgical removal of the underlying adenoma performed by an experienced neurosurgeon remains the cornerstone of treatment [5]. Most patients harbor macroadenoma and reported surgical cure rates vary widely [6]. Medical treatment is offered in persistent disease or as an alternative option to surgery in cases of contraindications or patient’s refusal [7]. Somatostatin analogs (SSAs), dopamine agonists (usually represented by cabergoline), and GH-receptor antagonists (pegvisomant) are currently used to battle acromegaly [7], while ongoing research focuses on new therapeutic agents and new administration routes for already available agents [8,9]. A third-line option is radiotherapy [10]; data on stereotactic radiosurgery seem to indicate that it is well tolerated and improves disease remission and control rates [11], but there is a noticeable delay between treatment and remission [12].

Although there are many treatment options available, a significant percentage of patients fail to achieve biochemical control of the disease [13]. Several factors (clinical, histological, radiological, and molecular) which can be interrelated seem to be associated with more aggressive tumor behavior and can play a prognostic role in acromegaly [14]. Pre-operative GH levels, invasiveness, and adenoma size seem to be associated with post-operative remission [15]. GH levels, granulation pattern, T2 magnetic resonance imaging (MRI) intensity, somatostatin receptor 2 (SSTR2), and E-cadherin staining can predict the level of response to first-generation SSAs but this information is not yet included in guidelines [16,17]. Low-T2-weighted signal in MRIs was initially reported to associate with better response to first-generation SSAs [18]. Furthermore, hypointense tumors usually display dense granulation as opposed to hyperintense tumors which tend to display sparse granulation and could be associated with lower levels of SSTR2 expression [16]. Evidence supports that pasireotide works better in a subset of patients who display the following characteristics: hyperintensity in T2 MRI, sparse granulation, low SSTR2, high SSTR5 expression, and low or mutated AIP, thus suggesting that pasireotide should be offered as first-line medical treatment under these conditions [17,19]. Currently, most patients receive first-generation SSAs in the context of a trial-and-error approach; however, there is an increasing need for personalized treatment [17,20] that will enable clinicians to offer patients the medical agents that best fit their individual profile of disease [21]. Pituitary tumors seem to develop in a tumor microenvironment (TME) composed of immune cells, cytokines, immune check-point molecules, and extracellular matrix [22]. The complex interplay between tumor cells and the TME may at least partially explain the observed heterogeneity in adenoma behavior that is translated in clinical outcomes [22]. Future research in the field of TME could offer new biomarkers and advance therapeutic progress by integrating targeted therapies such as immune check-point inhibitors, vascular endothelial growth factor (VEGF), and mammalian target of rapamycin (mTOR) inhibitors [22].

Acromegaly in the elderly is predicted to increase in the future and evidence, although sparse, seems to indicate that there are differences between younger and older patients [23]. The definition of elderly patients is not consistent in the literature; however, the usual cut-offs are 60, 65, or 70 years old [23]. Older patients with acromegaly tend to display a milder clinical phenotype, smaller tumor size, and better response to SSAs, while surgery is generally considered a safe option [23]. In one of the limited studies addressing surgical treatment in older versus younger patients, it was shown that despite the higher anesthesia risk and the higher incidence of cardiovascular disease and cancer, transsphenoidal surgery (TSS) is safe and also mitigates the comorbidities of older patients [24].

The aim of this study was to describe the journey of the disease towards treatment as depicted by the outcome of acromegalic patients who were followed up in an expert center for pituitary diseases and to analyze the clinical, biochemical, and pathological features of treated patients. A secondary aim was to describe an ill-defined unmet need regarding the features of elderly patients with acromegaly and to provide real-world data on the treatment approaches employed.

## 2. Methods

### 2.1. Patients

The patient medical records of patients with acromegaly visiting the outpatient clinic at Laiko Athens General Hospital in a timeframe from January 2005 to December 2024 were retrospectively reviewed. The Ethics Committee of Laiko General Hospital of Athens approved the protocol (2 April 2025 of approval and code 557/02-02-2024) and patient consent was obtained prior to the commencement of the study.

The retrieved information from patient records included age at diagnosis, IGF-1 levels at diagnosis, and onset of symptomatology, as recorded by the respective physician examining the patient each time. Second neoplasms, either benign or malignant, were also registered. The presence of comorbidities (type 2 diabetes, hyperlipidemia, hypertension, cardiovascular disease, hepatic steatosis, osteopenia, osteoporosis, and neoplasia) was recorded the first time it was documented in patients’ medical file. This could be either earlier to the diagnosis of acromegaly (as part of the patients past medical history) or later as recorded during follow-up or at the last visit. Hyperlipidemia was documented when LDL > 130 mg/dL and/or triglycerides > 150 mg/dL, or the patient received lipid-lowering medication [3,25,26]. Hypertension was documented when the patient had systolic blood pressure > 140 mmHg and/or diastolic plod pressure > 90 mmHg or received antihypertensive medication [26]. The retrieved information on adenoma size which was reported in pre-operative MRI was classified as microadenoma (<1 cm) or macroadenoma (≥1 cm). Granulation subtype (dense or sparse) as reported in available histology reports was also recorded. Initially, 83 patients with acromegaly were recorded. Four patients were excluded from the analysis, two of them were presented at the outpatient clinic only once with no further follow up, one of them was excluded due to ectopic GH excretion, and one of them was excluded because acromegaly was diagnosed in the context of multiple endocrine neoplasia syndrome.

Disease outcome was based on IGF-1 levels [1] as they were measured in blood samples using the method of chemiluminescence. The delay in diagnosis was estimated from the time of symptomatology onset up to the time of diagnosis. Diagnostic delay was from less than a year up to 35 years and was categorized into three groups. Group 0 describes patients who reported a delay of less than 5 years. Group 1 patients reported a delay of five to ten years, and group 2 patients reported a delay of ten or more years. These time intervals were selected as meaningful in regard to the existing literature since the average delay is reported to range from 2.9 to 5.5 years, while approximately 25% of patients wait for a decade or more before a diagnosis is established [27]. Longer delays translate into a higher disease burden for the patient as well as a higher financial burden for the healthcare system [27]. Patients were also divided into two groups according to their age at diagnosis. Group A refers to patients diagnosed with acromegaly before the age of 60 and group B to patients diagnosed at or after the age of 60.

### 2.2. Statistical Analysis

Collected data were analyzed using IBM SPSS Statistics 30.0.0 (1720). Numerical data were tested for canonical distribution using Shapiro–Wilk test and expressed in terms of mean ± standard deviation (SD) for normally distributed variables and median for non-normally distributed variables. Categorical data were expressed as frequencies and percentages.

For the correlations between continuous variables, Pearson correlation (r) was used if they had a normal distribution and Spearman (rho) was used if they did not have a normal distribution. For the associations between categorical variables, the chi-square test, Fisher’s exact test, and Fisher–Freeman–Halton exact test were used as appropriate. The T-test was applied to compare means for a variable between two independent groups of normally distributed data. The Mann–Whitney test was used to compare the distribution of a variable between independent groups for non-normally distributed data. The Kruskal–Wallis test was used to compare the means of three independent groups of unequal sample sizes. McNemars’ test was applied to compare difference in the proportion of a variable between two related groups. Binary logistic regression was used to calculate odds ratios. Each predictive factor in acromegaly outcome was tested separately using univariate logistic regression, but none of the parameters studied have shown a statistically significant *p*-value to perform a multivariate analysis. The binomial test and chi-square goodness-of-fit test were used to evaluate observed frequencies compared to equal frequencies. The *p*-value was set at 0.05.

## 3. Results

**Figure 1:**
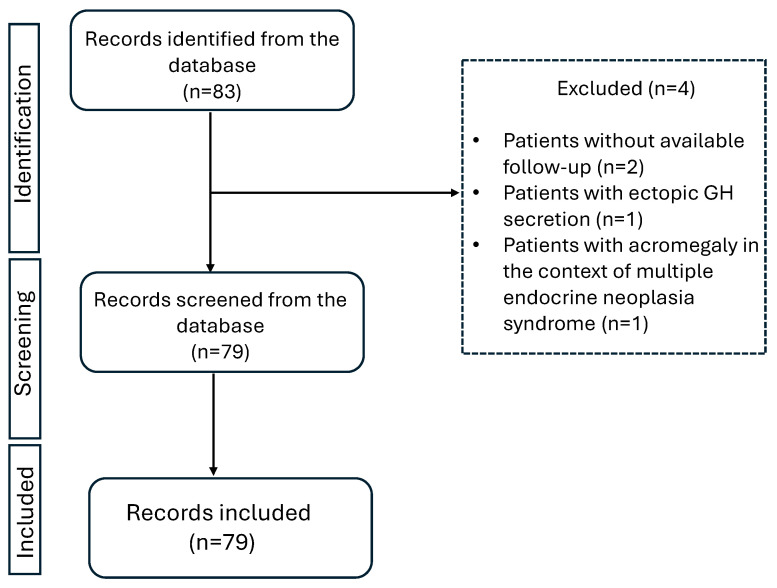
Study flowchart.

The study flowchart is shown in Figure 1. The dataset summarized in Table 1 consists of 79 patients diagnosed with sporadic acromegaly between 1971 and 2023. The median time from diagnosis to last follow-up was 8 years (range 1–42). In total, 43 patients (54%) were female. Overall, the mean age at diagnosis was 45 ± 13 years (range 17–76). The mean age at diagnosis for male was 45 ± 14 years while for female it was 45 ± 12 years. The mean age at diagnosis did not differ between male and female patients (*p*-value: 0.856).

Disease remission was documented in 67 out of 79 patients (85%, CI: 76.9–92.7%). Surgical remission was reported in 22 patients (28%, CI: 17.9–37.9%) while 12 patients out of 79 (15%, CI: 10.1–27.9%) failed to enter remission.

Surgery was offered to 57 patients, and 57 out of 79 patients (73%) underwent one procedure, 10 patients (13%) underwent a second surgery, and 11 out of 79 patients (14%) did not follow a surgical approach. In terms of medical treatment, 36 out of 54 patients (67%) were treated with one agent. The combination of two agents was documented in 14 out of 54 patients (26%), and 8 patients out of 14 (57%) received SSA plus GH receptor antagonist, while 6 patients out of 14 (43%) received SSA plus dopamine agonist. The use of three agents was reported in four patients (7%), in all these later cases patients received SSA with GH receptor antagonist and dopamine agonist. SSAs were used as monotherapy in 32 out of 36 patients (89%). SSAs used were octreotide long-acting repeatable or lanreotide autogel or pasireotide long-acting release. Dopamine agonists were used as monotherapy in 3 out of 36 patients (8%) and only one patient (3%) received GH receptor antagonist as monotherapy. Radiotherapy was used in 14 out of 79 patients (18%). A combination of treatment modalities was also recorded. In total, 33 patients out of 79 (42%) were offered surgery and drug therapy, and 8 patients out of 79 (10%) were offered surgery, drug therapy, and radiotherapy. Additionally, 4 patients out of 79 (5%) were offered surgery and radiotherapy, while 3 patients out of 79 (4%) were offered drug therapy and radiotherapy.

Overall, hyperlipidemia was documented in 35 out of 71 patients (49%), hypertension in 34 out of 72 patients (47%), type 2 diabetes in 29 out of 71 patients (41%), cardiovascular disease in 29 out of 72 patients (40%), hepatic steatosis in 20 out of 48 patients (42%), osteopenia in 12 out of 52 patients (23%), and osteoporosis in 11 out of 52 patients (21%). Regarding the time of comorbidity diagnosis, most patients were diagnosed after acromegaly diagnosis. Regarding the diagnosis time, before acromegaly diagnosis, three patients were diagnosed with type 2 diabetes, four with hyperlipidemia, two with hypertension, one patient with ischemic heart disease, two with cardiovascular disease (one with ischemic disease and one with both ischemic disease and diastolic dysfunction), and two patients with osteoporosis. The respective numbers after acromegaly diagnosis were 26 for type 2 diabetes, 31 for hyperlipidemia, 32 for hypertension, 27 for cardiovascular disease, and 9 for osteoporosis. Osteopenia and hepatic steatosis were all diagnosed after acromegaly diagnosis (Table 1). Regression logistic analysis showed that disease outcome (non-remission versus remission) did not affect the probability of being diagnosed with a comorbidity. However, type 2 diabetes showed a tendency to be predicted by disease outcome (OR: 4.136, CI: 0.97–76.633, *p*-value: 0.055) approaching statistical significance.

In total, 53 out of 76 patients (70%) developed, either before or after acromegaly diagnosis, another neoplasia. Overall, benign and malignant lesions developed in 49 (64%) and 15 (20%) patients, respectively. The type of neoplasia is described in detail in Table 2. Before acromegaly diagnosis, malignancies were reported in 4% of patients and increased four-fold post diagnosis to 16% (*p*-value: 0.035). The percentage of benign neoplasia was 20% before acromegaly diagnosis and 50% after acromegaly diagnosis (*p*-value: <0.001) (Table 2).

Regression logistic analysis showed that disease outcome (non-remission versus remission) did not affect the probability to develop a benign (OR: 2.805, CI: 0.699–11.252 *p*-value: 0.146) or malignant lesion (OR: 0, *p*-value: 0.999) before the diagnosis of acromegaly. Moreover, disease outcome did not affect the probability to develop a benign (OR: 0.321, CI: 0.078–1.321, *p*-value: 0.116) or malignant lesion (OR: 0.491, CI: 0.057–4.237, *p*-value: 0.518) after acromegaly diagnosis.

**Figure 2:**
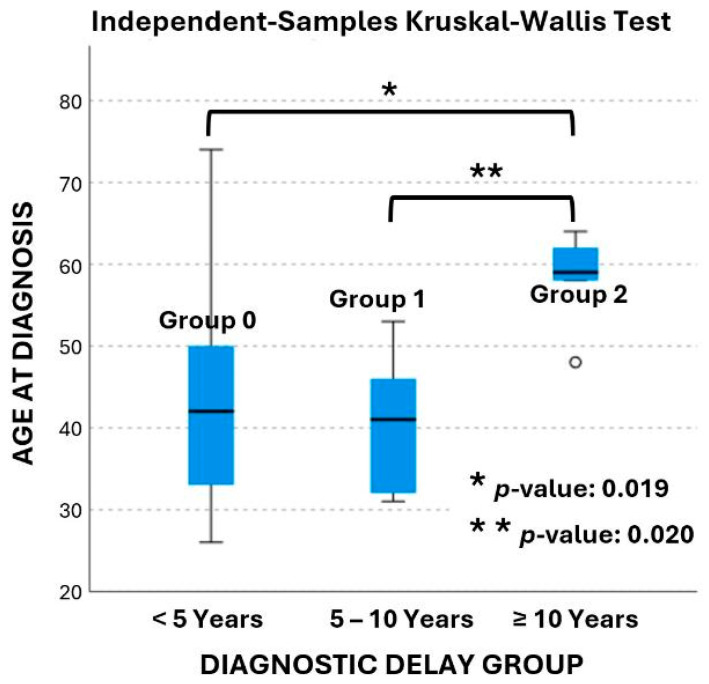
Box plot of age at diagnosis across the three delay groups.

In terms of the diagnostic delay, twenty-eight patients (65%) were in group 0, ten patients (23%) in group 1, and five patients (12%) in group 2. The age at diagnosis ranged from 26 to 74 years old and is presented in box plots in Figure 2. The mean age at diagnosis was 43 ± 12 years in group 0, 40 ± 8 years in group 1, and 58 ± 6 years in group 2, respectively. According to the data age was significantly different between groups (*p*-value: 0.015). Pairwise comparisons revealed that age at diagnosis was significantly different between groups 1 and 2 (*p*-value: 0.020), as well as between groups 0 and 2 (*p*-value: 0.019). The age at diagnosis was not statistically significant different between group 0 and group 1 (*p*-value: 1.0).

**Figure 3:**
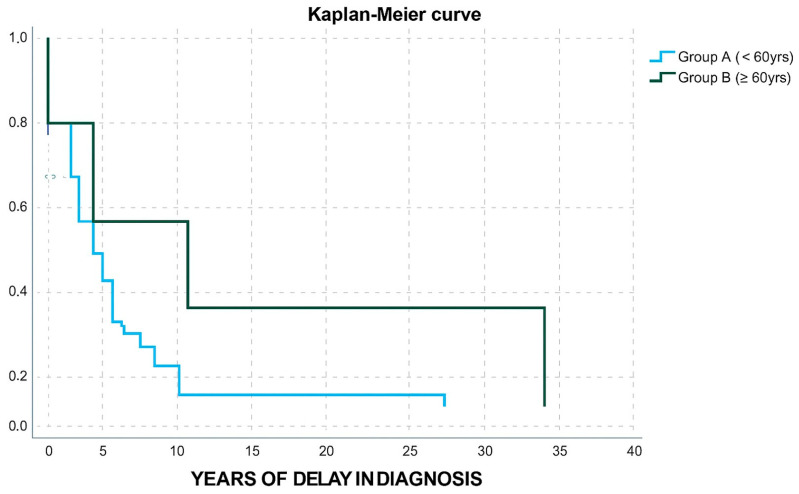
Kaplan–Meier curve for years of delay in diagnosis in group A and group B patients.

In terms of age, group A and B (Table 3) did not differ in gender distribution, IGF-1/ULN levels, adenoma size, granulation type, or disease outcome. Group B patients displayed a higher possibility to belong in group 2 of diagnostic delay (OR: 12, CI: 1.219–118.1, *p*-value: 0.033). Only one patient from group B received two medicines and only one patient from group B received a combination of three agents (OR: 0.518, CI: 0.096–2.795, *p*-value: 0.444). Moreover, only one patient from group B underwent radiotherapy (OR:0.364, CI: 0.043–3.077, *p*-value: 0.353); however, these results were not powered with statistical significance. Group B patients were less likely to undergo a surgical procedure (OR: 0.046, CI: 0.01–0.213, *p*-value < 0.001). A Kaplan–Meier curve was also used to demonstrate the difference in diagnostic delay between Group A and Group B (Figure 3).

Patient gender, age at diagnosis, IGF-1/ULN ratio at diagnosis, adenoma size, and granulation were assessed for their ability to predict non-remission in acromegaly using logistic regression analysis. According to the data (Table 4), IGF-1/ULN ratio at diagnosis shows a tendency to predict non-remission without reaching statistical significance, while the rest of the factors evaluated were not found to play a predictive role.

**Figure 4:**
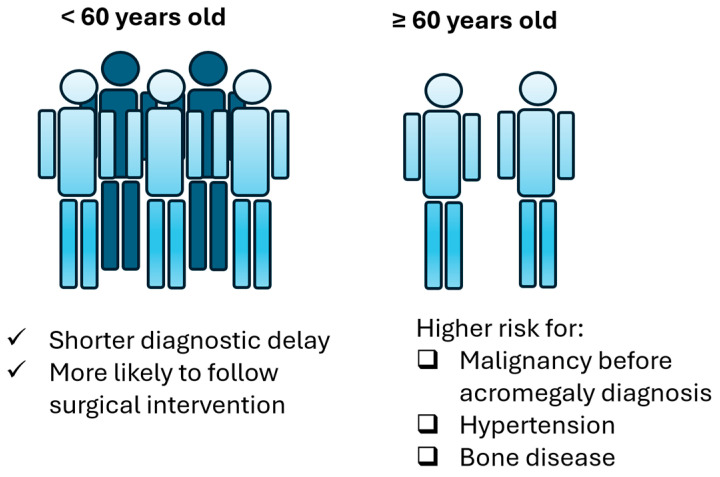
Main differences in acromegaly between group A and group B patients.

Regarding comorbidities (Table 3), group B patients display higher likelihood to present hypertension (OR: 6.48, CI: 1.289–32.578, *p*-value: 0.023) and bone disease (OR: 12.25, CI: 1.38–108.744, *p*-value: 0.025). Moreover, most patients presented one of the comorbidities after their diagnosis. Regression logistic analysis showed that older patients are not at increased risk of a malignancy diagnosis after acromegaly diagnosis (OR:1.222, CI: 0.229–6.517, *p*-value: 0.814) but they are at increased risk to develop a malignancy before acromegaly diagnosis (OR: 14.222, CI: 1.168–173.229, *p*-value: 0.037). Older age was not found to predict the possibility of developing benign lesions before (OR: 1.656, CI: 0.382–7.185, *p*-value: 0.5) or after acromegaly diagnosis (OR:0.808, CI: 0.224–2.914, *p*-value: 0.745). Collectively, the main differences between group A and group B patients are summarized in Figure 4.

## 4. Discussion

The present study has shown the increased rate of controlled patients with acromegaly followed up in one center with expertise in pituitary diseases, as opposed to previous studies [28,29,30,31]. The general demographic data presented in this study are in accordance with the literature, since acromegaly presents almost equally in male and female patients [32] and is diagnosed in the fifth decade of life [33], following an undiagnosed period of symptoms of about 5 years [33]. Macroadenomas are far more frequent than microadenomas, as previously described [6]. In alignment with a similar study from Greece, surgery is the treatment of choice for most cases and SSAs are the predominant medical option used [34]. Finally, diagnostic delay was more prominent in the elderly patients that had received a smaller number of treatments, and for whom being operated on was less possible.

Several factors are under investigation for their ability to influence and therefore predict outcome disease in acromegaly [14]. Recently, a scoring system was proposed to evaluate acromegaly outcome based on key factors including age, granulation, adenoma volume, increasing tumor size, baseline GH/IGF-1 levels, MRI T2 intensity, and ki67 [14]. In alignment with the literature, in this study, age at diagnosis, IGF-1/ULN ratio at diagnosis, adenoma size, and granulation were also selected to investigate their ability to predict non-remission versus remission in acromegaly patients. Even though younger patients usually present with more aggressive tumors compared to older patients [14], age at diagnosis is not a predictor of acromegaly outcome. Patient gender was also assessed in the current study as a meaningful candidate with potential impact on acromegaly outcome. In our dataset, gender does not seem to influence disease control as translated by age-adjusted IGF-1 levels. Meanwhile, there are conflicting results in the literature regarding the effect of gender on acromegaly control [35,36,37]. Of note, sex hormones affect GH function [37] and women display higher GH levels compared to men and seem to be vulnerable to disease complications even for the same level of biochemical control [38].

Micro- and macroadenomas did not associate with distinct outcomes; this is not surprising since a recent classification of acromegaly subtypes reports that type 1 acromegaly which holds the best prognosis is mainly attributed to non-aggressive densely granulated macroadenomas [39]. Meanwhile, type 2 tumors are macroadenomas of either dense or sparse granulation with an intermediate outcome and type 3 tumors are aggressive sparsely granulated macroadenomas with a poor biochemical outcome [39].

According to our results, a higher IGF-1/ULN ratio at diagnosis shows a tendency to associate with higher risk for non-remission. Higher baseline IGF-1 levels are associated with a higher risk for tumor growth in acromegaly [14]. Meanwhile, absolute IGF-1 values correlate with post-surgical biochemical control in short-term [6]. However, IGF-1/ULN ratio does not predict the level of biochemical control of the disease in the long term [6]. Future studies enrolling more patients could delineate the definitive impact of IGF-1/ULN ratio at diagnosis and sparse granulation in disease outcome.

Densely granulated adenomas are believed to display better prognosis compared to sparsely granulated [14], possibly due to distinct underlying mutations which influence therapeutic response to medical treatment [40]. However, according to our data, sparse granulation does not pose higher risk for non-remission in acromegaly. This is in accordance with another recent retrospective study which showed that the pathology subtype does not predict disease remission in acromegaly [41].

According to our data, patients reporting longer delays are older at the time of diagnosis, which could be explained by the fact that younger patients harbor more aggressive adenomas and are diagnosed earlier [39]. This finding could imply that older patients have milder diseases with slower progression. Moreover, aging can overshadow the characteristic changes in the disease, making it more difficult to identify them [42]. As a result, this finding should raise awareness among clinicians to stay alert for the insidious symptoms of acromegaly in the elderly. In addition, the therapeutic approach is found to differ between younger and older patients. Only one patient over the age of 60 underwent radiotherapy. Similar results are reported in a retrospective Italian study, where patients over the age of 65 did not receive radiotherapy [43]. The most probable rationale explaining this finding is the long timeframe needed for this type of treatment to produce results accompanied by a high risk of potential side-effects [43]. Interestingly, older patients are also less likely to be surgically treated. This result is reminiscent of a nation-wide analysis in Spain regarding elderly patients with acromegaly, where almost 30% of patients refused surgery, 10% of them where not fit for surgery, and some patients refused any treatment [44]. Older acromegalic patients display lower quality of life, and this group of patients is expected to increase in the future [45]. Therefore, future studies trying to delineate the optimal approach in this subpopulation of patients will greatly benefit patient care.

Of note, when interpreting our results, we should acknowledge how acromegaly treatment has evolved throughout the years. Bromocriptine, a dopamine agonist, was found to reduce GH secretion in the 1970’ [46], followed by octreotide in the mid-eighties, which was reported to be effective in cases of acromegaly where surgery and/or radiotherapy failed [47]. In the late 1990s, another dopamine agonist called cabergoline showed promising results [48]. Pegvisomant was introduced as a novel agent battling acromegaly in 2000 [49], while in 2014, pasireotide received approval by the European Medicine Agency for acromegaly [50]. The sequence of therapeutic approaches has also been subjected to refinement, reflecting the ongoing advances in the field. For this reason, leading authorities and organizations regularly publish updated guidelines [5,51,52,53,54,55], while the rising needs to offer personalized treatment [20] and a multidisciplinary approach [54] are being increasingly recognized.

Comorbidities’ management on acromegaly has been recently revised [55]. The frequency of comorbidities recorded in the study is as high as expected, since conditions such as hypertension, diabetes, and dyslipidemia are known to be more common in patients with acromegaly compared to the general population, and most comorbidities presented after diagnosis [56]. Similarly, osteoporosis incidence is comparable to previously published results [56,57,58]. Regarding neoplasia, this study shows higher incidence after the diagnosis of acromegaly, with thyroid and breast cancer being the most frequent malignancies observed. Several studies showed that thyroid cancer is the most frequent malignancy [56,59,60] and breast cancer is among the top three [56]. In accordance with the literature [56], in our study thyroid nodules are the most frequent benign lesions, followed by colon adenomas. Our results also demonstrate a high prevalence of adrenal lesions, as previously described [61].

The risk for cancer in the context of acromegaly is debatable [62]. There is evidence that acromegaly is accompanied by a higher risk for both benign and malignant neoplasia (s) but whether this is a consequence of the disease or a result of more intense screening remains to be determined [63]. Although there are no guidelines on the appropriate screening, emphasis is given to deciding according to individual risk factors, taking into consideration the burden of uncontrolled disease [64]. Despite the uncertainty, it seems likely that patients with acromegaly are at higher risk for colorectal cancer and leading authorities in the field advise colonoscopy screening [65]. Overall, acromegaly possibly carries a higher risk for both benign and malignant neoplasms (particularly colorectal and thyroid), especially in patients with untreated or poorly controlled disease, once again emphasizing the need for early detection and proper disease management [66]. It is of note that the outcome did not affect the presence of second neoplasms after acromegaly diagnosis.

This study shows that elderly acromegaly patients are at higher risk of developing malignancy before acromegaly diagnosis as well as bone disease and hypertension. Older age in acromegaly [67,68] as well as positive family history [68] were previously described as risk factors for cancer. Acromegaly is known as a cause of secondary osteoporosis [69]. Excess GH is associated with high bone turnover and disrupted bone architecture, thus leading to fractures even in the presence of normal bone density [23]. Vertebral fractures are increasingly recognized as one of the most common disease complications observed in acromegaly [70], and, at least in the general population, mortality rate is high for vertebral fractures in patients over 65 years old [71]. Elderly acromegaly patients also display higher blood pressure levels compared to age-matched controls [72], while older acromegaly patients seem to be at higher risk for hypertension, left ventricular hypertrophy, and metabolic disorders [73]. The results from the ACROSTUDY indicate that older age is associated with hypertension, which carries a higher mortality risk particularly in the presence of cardiovascular disease [26]. Collectively, this vulnerable subset of patients presents with comorbidities that can adversely affect both life expectancy and quality, thus underscoring the value of age in this disease and therefore directing clinical attention towards individualized care pathways [55].

There are several limitations in the present study. Firstly, this is a single-center retrospective study and as a result only few patients with this rare disease were recruited and were almost all subjected to similar diagnostic and therapeutic protocols. The number of patients is relatively small, but this is explained given the rarity of the disease, the small population of Greece, and the fact that not all acromegalic patients are referred to centers with expertise. Moreover, given the nature of the study, there could be referral bias, and it should be mentioned that there could be heterogeneity in laboratory assays throughout the years. Lastly, the lack of age-matched control group in the study is another limiting factor in the interpretation of comparison between groups.

## 5. Conclusions

Biochemical control rate in acromegaly is satisfactorily high in a center with expertise to treat these conditions. Surgical removal of the adenoma is offered in most patients and SSAs are the most frequently used agents. Demographic factors are not found to predict disease outcome; however, the IGF-1/ULN ratio at diagnosis shows a tendency to predict acromegaly outcome. Acromegalic patients display high frequency of metabolic disorders, hypertension, and neoplasia, highlighting once again the need for close monitoring and adherence to preventive strategies by a multidisciplinary team targeting in a personalized management. Older patients are diagnosed with longer delays and are less likely to follow interventional treatment. Older acromegalic patients display a higher risk of developing a malignancy before acromegaly diagnosis as well as hypertension and bone disease as opposed to younger acromegalic patients. Overall, this study reveals that the age of diagnosis can influence several aspects of acromegaly, from diagnosis to treatment and comorbidities, thus indicating the importance and impact of age in this rare condition.

## Figures and Tables

**Table 1:** Dataset overview.

Category	Subcategory	Number of Patients (at Diagnosis)	Percentage of Patients	*p*-Value
Gender	Female	43	54%	0.500 ^1^
	Male	36	46%	
Age group	17–39	30	38%	<0.001 ^2^
	40–59	37	47%	
	≥60	12	15%	
Outcome	Remission	67	85%	<0.001 ^1^
	Non remission	12	15%	
Surgery	No surgery	11	14%	<0.001 ^2^
	One surgery	57	73%	
	Two surgeries	10	13%	
Medical agents	One agent	36	67%	<0.001 ^2^
	Two agents	14	26%	
	Three agents	4	7%	
Monotherapy	*SSAs	32	90.3%	<0.001 ^1^
	Dopamine agonists	3	9.7%	
	Pegvisomant	1	3%	
Radiotherapy	No	63	82%	<0.001 ^1^
	Yes	14	18%	
Adenoma size	Microadenoma	8	12%	<0.001 ^1^
	Macroadenoma	58	88%	
Granulation	Dense	14	47%	1.000 ^1^
	Sparse	16	53%	
Type 2 diabetes ^3^	No	42	59%	0.154 ^1^
	Yes	29 (3)	41%	
Hyperlipidemia ^4^	No	36	51%	1.00 ^1^
	Yes	35 (4)	49%	
Hypertension ^5^	No	38	53%	0.724 ^1^
	Yes	34 (2)	47%	
Cardiovascular disease ^6^	No	43	60%	0.125 ^1^
	Yes	29 (2)	40%	
Cardiovascular disease	Valvular lesions	19	26%	-
	Ischemic disease	4 (2)	5.5%	
	Hypertrophy	6	8%	
	Diastolic dysfunction	13 (1)	18%	
	Systolic dysfunction	3	4%	
Hepatic steatosis ^7^	No	28 (0)	58%	0.312 ^1^
	Yes	20 (0)	42%	
Bone disease	No	29	56%	0.488 ^1^
	Yes	23 (2)	44%	
Osteopenia/Osteoporosis ^8^	-	12/11 (2)	23%/21%	-

^1^ Binomial test. ^2^ Chi-square goodness-of-fit test. ^3^ Diagnostic criteria: fasting plasma glucose ≥ 126 mg/dL more than once, two-hour plasma glucose ≥ 200 mg/dL after 75 g oral glucose test, or random plasma glucose ≥ 200 mg/dL with symptomatic hyperglycemia. ^4^ Diagnosed when LDL > 130 mg/dL or triglycerides > 150 mg/dL or lipid-lowering medication used. ^5^ Diagnosed when systolic blood pressure > 140 mmHg and/or diastolic plod pressure > 90 mmHg or antihypertensive medication used. ^6^ Ischemic disease was diagnosed according to patients’ medical history of myocardial infarction or based on heart scintigraphy. Valvular lesions, hypertrophy, diastolic, and systolic dysfunction were diagnosed based on heart ultrasounds. ^7^ Diagnosed in imaging (abdomen ultrasound or computed tomography scan CT or MRI). ^8^ Osteopenia was diagnosed when t-score was between −1 and −2.5 in lumbar spine or left/right hip using bone densitometry. Osteoporosis was diagnosed when t-score was ≤−2.5 in lumbar spine or left/right hip using bone densitometry. () Number in parenthesis indicates the number of patients with a diagnosed comorbidity before acromegaly diagnosis. *SSAs used were octreotide long-acting repeatable or lanreotide autogel or pasireotide long-acting release.

**Table 2:** Neoplasia in acromegaly.

Neoplasia Before or at the Time of Acromegaly Diagnosis			
Benign		Malignant	
Neoplasia	Number of patients (percentage)	Neoplasia	Number of patients (percentage)
Thyroid nodules	7 (10%)	Papillary thyroid carcinoma	1 (1.4%)
Colon tubular adenoma, polyp	1 (1.3%), 2 (2.6%)	Bladder carcinoma	1 (1.4%)
Ovarian teratoma	1 (1.3%)	Breast cancer	1 (1.4%)
Lipoma	1 (1.3%)	-	-
Endometrial hyperplasia	1 (1.3%)	-	-
Cervix polyp; vocal cord polyp	1 (1.3%), 1 (1.3%)	-	-
**Neoplasia after the time of acromegaly diagnosis**			
Benign		Malignant	
Neoplasia	Number of patients (percentage)	Neoplasia	Number of patients (percentage)
Thyroid nodules	26 (34%)	Papillary thyroid carcinoma	3 (3.9%)
Colon adenoma; polyp	6 (8%), 8 (10.5%)	Breast cancer	3 (3.9%)
Adrenal adenoma–hyperplasia	13 (17%)	Kidney cancer	2 (2.6%)
Meningioma	2 (2.6%)	Prostate cancer	1 (1.3%)
Bladder papilloma	1 (1.3%)	Urothelial cancer	1 (1.3%)
Esophagus papilloma	1 (1.3%)	Colon cancer	1 (1.3%)
Uterine fibromyoma	1 (1.3%)	Melanoma	1 (1.3%)
Other (pancreatic lesion, kidney mass, or ECL hyperplasia)	3 (3.9%)	Duodenal neuroendocrine tumor	1 (1.3%)
-	-	Paraganglioma	1 (1.3%)

**Table 3:** Acromegaly characteristics in group A (diagnosed before the age of 60 years) and group B (diagnosed at or above the age of 60 years) patients.

Category	Subcategory	Group A (<60 yrs)	Group B (≥60 yrs)	OR (CI)	*p*-Value
Gender	Female	37 (55%)	6 (50%)	1.233 (0.361–4.219)	0.738 ^1^
	Male	30 (45%)	6 (50%)		
Diagnosis age; mean ± SD	-	41 ± 10	65 ± 5	-	<0.001 ^2^
IGF-1/ULN ratio; mean ± SD/median	-	2.6 ± 1/2.3	3.5 ± 2/3	-	0.434 ^2^
Adenoma size	Microadenoma	6 (11%)	2 (18%)	0.551 (0.096–3.174)	0.505 ^1^
	Macroadenoma	49 (89%)	9 (82%)		
Granulation	Dense	13 (46%)	1 (50%)	0.867 (0.049–15.279)	0.922 ^1^
	Sparse	15 (54%)	1 (50%)		
Outcome	Remission	58 (87%)	9 (75%)	2.148 (0.487–9.469)	0.312 ^1^
	Non remission	9 (13)	3 (25%)		
Diagnostic delay group	0 (<5 years)	26 (66.7%)	2 (50%)	12 (1.219–118.1) ^3^	0.033 ^1^
	1 (5–10 years)	10 (25.6%)	0		
	2 (≥10 years)	3 (7.7%)	2 (50%)		
Surgery	No surgery	4 (6%)	7 (58%)	0.046 (0.01–0.213) ^4^	<0.001 ^1^
	1 surgery	52 (79%)	5 (42%)		
	2 surgeries	10 (15%)	0		
Medical agents	One agent	29 (64%)	7 (78%)	0.518 (0.096–2.795) ^5^	0.444 ^1^
	2 agents	13 (29%)	1 (11%)		
	3 agents	3 (7)	1 (11%)		
Radiotherapy	No	52 (80%)	11 (92%)	0.364 (0.043–3.077)	0.353 ^1^
	Yes	13 (20%)	1 (8%)		
Cardiovascular disease	-	22 (36%)	7 (64%)	3.102 (0.816–11.789)	0.096
Type 2 diabetes	-	23 (38%)	6 (54.5%)	1.93 (0.528–7.054)	0.320
Hyperlipidemia	-	29 (48%)	6 (54.5%)	1.283 (0.353–4.661)	0.705
Hypertension	-	25 (41%)	9 (82%)	6.48 (1.289–32.578)	0.023
Hepatic steatosis	-	18 (46%)	2 (18%)	0.333 (0.061–1.812)	0.203
Bone disease	-	16 (36%)	7 (87.5%)	12.25 (1.38–108.744)	0.025
Osteopenia	-	8 (18%)	4 (50%)	4.5 (0.924–21.925)	0.063
Osteoporosis	-	8 (18%)	3 (37.5%)	2.7 (0.532–13.691)	0.230

^1^ Binary logistic regression. ^2^ Mann–Whitney test. ^3^ Diagnostic delay of ≥10 years versus <10 years. ^4^ Surgery versus no surgery. ^5^ More than one medicine versus one medicine.

**Table 4:** Predictive factors in acromegaly outcome.

Variable	Exp (B)	95% CI for Exp (B)	*p*-Value
Gender (male vs. female)	1.233	0.361–4.219	0.738
Age at diagnosis	1.016	0.95–1.087	0.647
IGF-1/ULN ratio at diagnosis	1.717	0.903–3.264	0.099
Adenoma size	1.286	0.141–11.75	0.824
Granulation type (sparse vs. dense)	1.385	0.196–9.768	0.744

## Data Availability

The original contributions presented in this study are included in the article. Further inquiries can be directed to the corresponding author.

## Abbreviations

The following abbreviations are used in this manuscript:

CI	Confidence interval
CT	Computed tomography scan
GH	Growth hormone
IGF-1	Insulin-like growth factor-1
MRI	Magnetic resonance imaging
mTOR	Mammalian target of rapamycin
OR	Odds ratio
SD	Standard deviation
SSAs	Somatostatin analogs
SSTR2	Somatostatin receptor 2
TME	Tumor microenvironment
TSS	Transsphenoidal surgery
ULN	Upper limit of normal
VEGF	Vascular endothelial growth factor
